# Ultrasound-Guided Thoracic Paravertebral Injection of Platelet-Rich Plasma for the Treatment of Thoracic Herpes Zoster-Related Pain: A Study Protocol

**DOI:** 10.1007/s40122-024-00691-6

**Published:** 2024-12-12

**Authors:** Liu Wang, Xinyu Lei, Zhixuan Lan, Ruilin He, Zongbin Jiang

**Affiliations:** Department of Pain Medicine, The Second Affiliated Hospital of Guangxi Medical University, Nanning, 530007 Guangxi China

**Keywords:** Herpes zoster-associated pain, Ultrasound-guided, Platelet-rich plasma, Antiviral therapy

## Abstract

**Background:**

Herpes zoster (HZ), triggered by the reactivation of the varicella-zoster virus, manifests as a painful rash known as zoster-associated pain (ZAP), which can progress to postherpetic neuralgia (PHN). This study evaluates the efficacy and safety of ultrasound-guided thoracic paravertebral injections of platelet-rich plasma (PRP) in managing acute ZAP and preventing PHN.

**Methods:**

This is a prospective, randomized, controlled, open-label, endpoint-blinded, single-center trial involving 128 participants suffering from zoster-associated pain. Participants will be randomly assigned to the PRP treatment in combination with antiviral therapy group or the antiviral therapy group at a 1:1 ratio. Pain intensity (NRS-11), quality of life (SF-12), sleep quality (PSQI), pain characteristics, skin lesion recovery, average weekly consumption of rescue analgesics, and adverse events will be assessed. Follow-up assessments will be conducted at 1, 3, 6, and 12 months post-intervention to evaluate the incidence rate of PHN, pain intensity, quality of life, sleep quality, and safety.

**Ethics and Dissemination:**

Adhering to the 2013 SPIRIT statement and the Declaration of Helsinki, this study has received ethical approval from the relevant committee. Results will be disseminated through scientific journals and conferences, contributing to global data on managing ZAP.

**Conclusions:**

By comparing PRP with antiviral therapy, this trial seeks to establish a more effective treatment paradigm for reducing acute zoster-associated pain and the incidence of PHN, potentially setting a new standard in therapeutic strategies for HZ.

**Trial Registration:**

This clinical trial is registered with the Chinese Clinical Trial Registry (ChiCTR) at https://www.chictr.org.cn/index.html (Registration Number: ChiCTR2400087248, Registration Date: 2024–07-23).

## Key Summary Points

**Table:** 

***What did the study ask?***
This study aims to assess the effectiveness and safety of platelet-rich plasma (PRP) injections, comparing it with standard antiviral therapy to reduce zoster-associated pain (ZAP) and prevent postherpetic neuralgia (PHN).
***What was learned from the study?***
Expected outcomes include insights into the impact of PRP on managing acute ZAP and potentially establishing PRP as an effective adjunctive therapy to antiviral treatments in preventing PHN.
The study may pave the way for new treatment strategies in herpes zoster (HZ)-related pain management, especially if PRP is shown to improve long-term outcomes.

## Background

Herpes zoster (HZ), commonly known as shingles, is a painful dermatological condition triggered by the reactivation of the varicella-zoster virus (VZV). Approximately 30% of individuals are at risk of developing HZ over their lifetime if not vaccinated [1]. This virus resides in a dormant state within the dorsal root or trigeminal ganglia in adults. Its reactivation induces acute neuroinflammation and subsequent long-term damage to neurons and supportive cells, resulting in persistent pain known as zoster-associated pain (ZAP). Postherpetic neuralgia (PHN) is conventionally defined as pain in the affected dermatome that persists for at least 3 months after the healing of the acute herpes zoster rash [2]. Patients over the age of 50 are more likely to develop HZ [3, 4]. PHN can significantly impact patients' quality of life, causing persistent pain that interferes with daily activities and sleep.

Antiviral treatment is the cornerstone of HZ management, aiming to reduce viral replication, accelerate lesion healing, and alleviate acute pain. Common antiviral agents include acyclovir, valacyclovir, and famciclovir, which inhibit viral DNA synthesis. Despite their efficacy in reducing acute pain and viral shedding, antiviral medications have limitations. They do not completely prevent the development of PHN, particularly in patients with severe initial pain, older age, combined with underlying diseases or compromised immune systems [5, 6]. The partial efficacy of antivirals underscores the need for adjunctive therapies to more effectively manage ZAP and prevent its progression to PHN [7]. Studies highlight the thoracic region as the most commonly affected area [8].

Platelet-rich plasma (PRP) is a preparation made by extracting and enriching platelets from the patient's own blood. PRP contains high concentrations of growth factors and cytokines, which promote tissue repair, angiogenesis, and anti-inflammatory effects. The mechanisms of PRP include the release of bioactive molecules such as platelet-derived growth factor (PDGF), transforming growth factor-beta (TGF-β), and vascular endothelial growth factor (VEGF), which enhance tissue healing and reduce inflammation. Recent advances suggest that PRP can improve outcomes in various medical conditions, including chronic wounds, musculoskeletal injuries, and dermatological disorders [9–11]. PRP has been demonstrated to possess nerve repair and pain relief properties and has been studied in the treatment of both peripheral and central nervous system injuries, including the facial nerve [12–14], median nerve [15, 16], sciatic nerve [17, 18], and spinal cord [19, 20]. Although there is limited direct evidence supporting PRP’s role in preventing PHN, its regenerative and anti-inflammatory properties indicate its potential in managing ZAP and reducing the risk of PHN.

Given the limitations of current treatments and the potential benefits of PRP, our study aims to evaluate the additional effectiveness of PRP combined with standard antiviral therapy compared to antiviral treatment. To date, no comprehensive, long-term follow-up studies have evaluated the effect of PRP on ZAP. Therefore, a prospective, randomized study is needed to compare the effects of PRP on ZAP management and the prevention of PHN. This could provide a simple and feasible strategy for controlling ZAP and preventing PHN. Cadaver studies have demonstrated that under ultrasound guidance, thoracic paravertebral intervertebral injections encompassed the thoracic nerve root, reached the intervertebral foramen, and a spread is observed within the intercostal space, saturating the intercostal nerve [21–23]. Therefore, our research has decided to select ultrasound-guided thoracic paravertebral nerve injection technique as our fundamental technique for this study, comparing PRP injection therapy (experimental group) with antiviral therapy alone (control group).

## Methods

### Study Objectives

The primary objective of this study is to assess the effectiveness of ultrasound-guided thoracic paravertebral PRP injections in alleviating acute ZAP and preventing the onset of PHN. The secondary objective is to compare the outcomes of PRP injections with traditional antiviral therapy in treating acute HZ.

### Study Design and Recruitment

This study is a prospective, randomized, controlled, open-label, endpoint-blinded single-center trial. We plan to recruit 128 patients with ZAP at the Second Affiliated Hospital of Guangxi Medical University. Before the start of the study, all researchers will receive standardized training, including trial content, treatment strategies, treatment methods, evaluation and quality control, etc., and pass the assessment. Patients will be randomly assigned to the thoracic paravertebral PRP injection group or the antiviral therapy group at a 1:1 ratio. Recruitment will be conducted by experienced attending physicians.

**Fig. 1:**
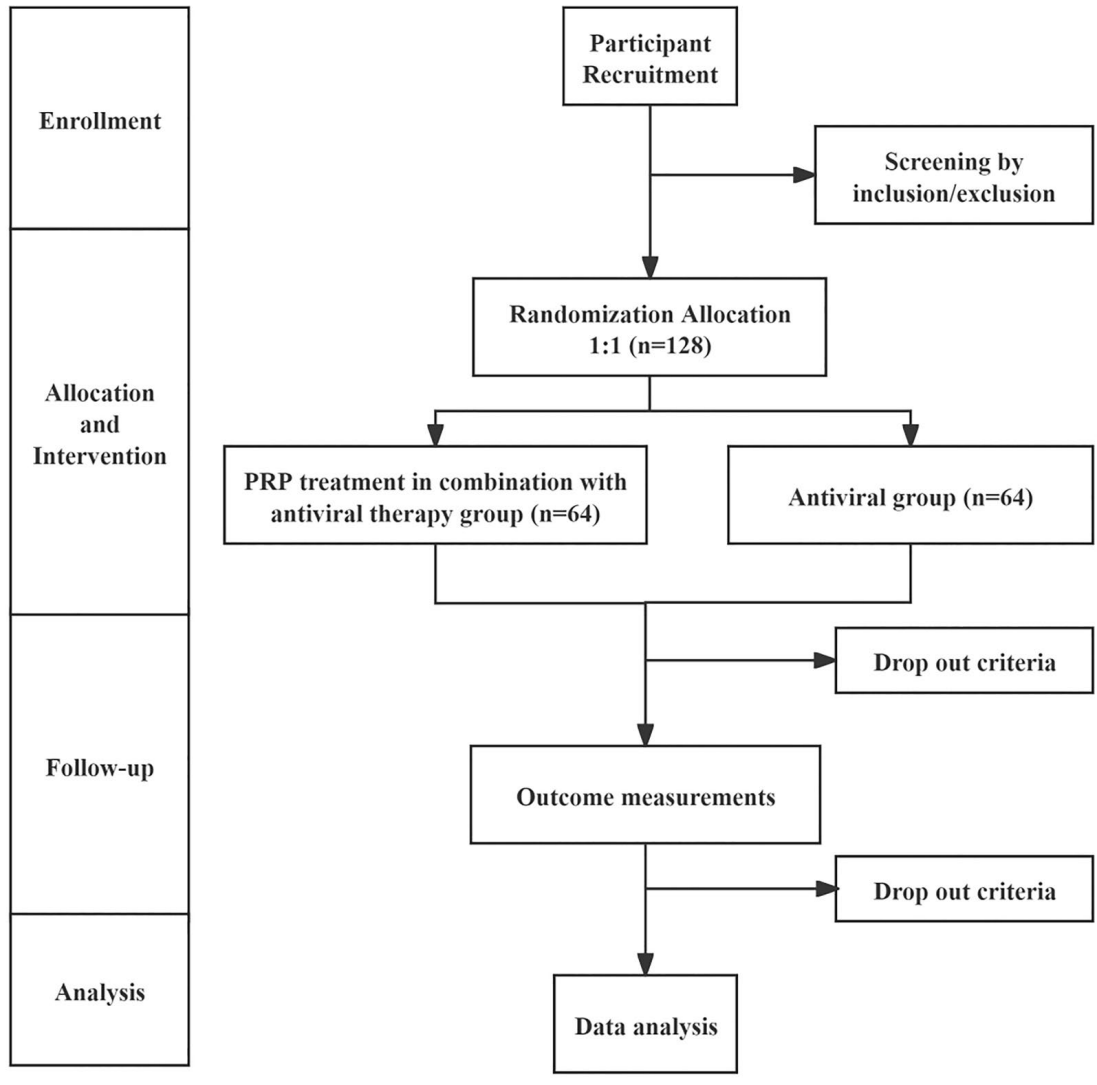
Trial flow diagram. *PRP* platelet-rich plasma

All potential study participants will be provided with a detailed understanding of the study purpose, interventions, potential benefits, possible risks, and countermeasures, and will have at least 1 h to consider whether to join the study. They will be rigorously evaluated according to the inclusion and exclusion criteria. Eligible patients will be enrolled in the study and sign an informed consent form, with the right to withdraw from this study at any time. The confidentiality of participant records will be protected. The patient flow diagram of the clinical trial is illustrated in Fig. 1

#### Inclusion Criteria

1. Diagnosed with HZ involving unilateral thoracic nerves (T1–T12).
2. Age ≥ 50 years and disease duration ≤ 2 weeks.
3. Average pain intensity score (11-point NRS) within 24 h before randomization ≥ 4 points.
4. Agree to participate in this study and be able to sign the informed consent form.

#### Exclusion Criteria

1. Patients with disseminated HZ or combined with HZ at other locations.
2. Coagulation dysfunction, taking anticoagulant drugs or/and antiplatelet drugs.
3. Systemic infections or infection at the puncture site.
4. Pregnant or lactating women.
5. Patients with immunodeficiency diseases, severe cardiovascular, cerebrovascular, and respiratory diseases, or other serious systemic diseases that prevent cooperation with the treatment.
6. Patients with psychiatric conditions.
7. Patients with thoracic spine deformity or a history of thoracic spine surgery that affects ultrasound positioning.
8. Patients who have contraindications for PRP or are allergic to treatment-related drugs such as famciclovir, gabapentin, pregabalin, nonsteroidal anti-inflammatory drugs (NSAIDs), and tramadol.
9. Patients with a recent history of other pain management treatments

### Case Dropout Instructions

1. If a subject develops other comorbidities, complications, or special physiological changes during the trial and is deemed unfit to continue, they will be classified as a case dropout.
2. If a subject encounters a serious or key adverse event during the trial, and our research team judges it inappropriate to continue, the subject will be withdrawn.
3. If a subject adds medications not specified in the protocol or receives external treatment, it will be considered a protocol violation, and they must be removed from the trial.
4. Subjects may choose to withdraw due to personal reasons or be lost to follow-up in the final stage of the trial.

For all cases of dropout, our research team will actively complete the last follow-up and examination to collect data and analyze effectiveness and safety. Detailed information on all dropout cases, including test results and reasons for dropout, will be fully documented on the case report form.

### Randomization and Blinding

Participants will be randomly assigned to receive either PRP treatment in combination with antiviral therapy or antiviral therapy alone in a 1:1 ratio using block randomization with a block size of 4, created with IBM SPSS Statistics 25.0. This sequence will be secured in numbered, opaque envelopes to ensure allocation concealment. Treatment efficacy and safety evaluations will be conducted by independent assessors unaware of the group assignments.

Due to the nature of the interventions, complete blinding is not possible for the doctors and patients. Therefore, this study will use blinded endpoints. The efficacy and safety evaluations will be performed by personnel not directly involved in the treatment. The evaluators who perform the assessments and the personnel conducting the statistical analysis will be blinded to the group assignments.

### Interventions

#### PRP Preparation

In this study, we employed a classical two-step centrifugation method to prepare 3 ml of PRP for paravertebral injection therapy. The PRP used in this study is classified as pure PRP (P-PRP), which contains a high concentration of platelets but minimal leukocytes, following the classification by Dohan Ehrenfest et al.[24].

1. Blood collection: Approximately 30 ml of venous blood is drawn from each patient using sodium citrate as an anticoagulant to inhibit coagulation.
2. First centrifugation: The collected blood undergoes a first centrifugation at room temperature at low speed (160–200 g) for 15 min, resulting in a separation into three distinct layers: a lower red blood cell layer, a middle buffy coat rich in white blood cells, and an upper plasma layer with a low platelet count.
3. Platelet enrichment: The upper layer of plasma, excluding the white blood cell-rich buffy coat, is gently transferred to a new sterile centrifuge tube, taking care to avoid disturbing the middle layer.
4. Second centrifugation: This plasma is then centrifuged at a higher speed (500–600 g) for 10 min to further concentrate platelets at the bottom of the tube.
5. PRP collection: After centrifugation, the upper plasma is carefully aspirated, leaving about 4 ml of PRP at the bottom of the tube. From this, 1 ml is used for platelet counting and activity analysis to ensure the prepared PRP meets clinical standards, and the remaining 3 ml is utilized for ultrasound-guided paravertebral injections.

#### Ultrasound-Guided Thoracic Paravertebral Injection Technique

We adopted an ultrasound-guided approach for thoracic paravertebral injection, as detailed in Pu et al. [25]. The patient will be placed in the prone position to optimize access to the thoracic paravertebral space. A low-frequency convex array ultrasound probe (2–5 MHz) will be used to localize the target thoracic levels, starting in the paraspinal sagittal position and moving caudally to identify the ribs. After identifying the first rib, the probe will be moved further down the thoracic spine to locate the target segments. Typically, one to three thoracic levels will be selected based on the dermatomal distribution of ZAP, with emphasis on the most affected segments (e.g., T3, T4, and T5 for T3–T5 involvement).

Once the target level is identified, the probe will be rotated 90 degrees to achieve an axial view, allowing visualization of the transverse process, inferior articular process (IAP), and parietal pleura (PP). A 22-gauge, 80–100-mm echogenic needle will be inserted in-plane from lateral to medial, aiming for the midpoint between the IAP and PP. Doppler ultrasound will be used to avoid puncturing vascular structures. After confirming the needle placement, 3 ml of PRP will be injected at each targeted level under continuous ultrasound guidance.

Following the injection, patients will be monitored for 30 min to observe any adverse effects. A follow-up ultrasound will be performed to verify the distribution of the injectate and exclude complications such as hematomas or pleural punctures.

#### Treatment Interval and Treatment Dose

Patients in both groups will receive standard antiviral therapy with famciclovir 500 mg 3 times daily for 7 days, initiated immediately upon recruitment during the viral outbreak [26]. In addition to the standard antiviral therapy, patients in the experimental group will receive PRP injections. The interval between each ultrasound-guided thoracic paravertebral injection is one week, with a total of four injection treatments. Each injection includes 3 ml of PRP per affected thoracic nerve.

#### Additional Intervention

Antiviral therapy has been widely used as the standard treatment for HZ [27]. During the treatment process, pain management will be adjusted based on pain severity and nature of the pain. For mild pain, non-opioid analgesics, such as NSAIDs, will be administered, with opioids added if the pain progresses to moderate or severe levels. Given the neuropathic component of ZAP, tricyclic antidepressants or anticonvulsants, such as gabapentin or pregabalin, may also be incorporated to enhance pain relief [28]. If the ultrasound-guided PRP injection is unsuccessful or the treatment cannot be completed for any other reason, the corresponding patient will be considered a protocol violation and must be excluded from the study.

After completing the study, the doctor will decide whether to continue auxiliary medications or other treatments based on the patient's specific condition. The dosage of medication will be adjusted according to changes in the patient's pain level, aiming to optimize therapeutic effects and improve quality of life. Treatments not mentioned in this protocol will not be permitted unless approved and standardized by the research team. All additional treatments will be documented in detail by the research assistant.

### Follow-up and Participant Timeline

After patients receive therapy, systematic follow-up will be conducted. Follow-up time points are shown in Table 1.

**Table 1:** Timeline of participant enrolment, allocation, interventions, and outcome assessments

Time point	Enrolment	Allocation	Post-allocation								Close out
	D0	D0	w1	w2	w3	w4	M1	M2	M3	M6	M12
Enrolment											
Eligibility screening	×										
Informed consent	×										
Allocation		×									
Intervention											
PRP treatment			×	×	×	×					
Antiviral therapy			×								
Assessment											
Demographics	×										
Incidence rate of PHN									×	×	×
NRS-11	×	×	×	×	×	×	×	×	×	×	×
Pain characteristics	×	×	×	×	×	×	×	×	×	×	×
Skin lesions	×	×	×	×	×	×	×	×	×	×	×
SF-12		×				×			×	×	×
PSQI		×				×			×	×	×
Analgesic consumption			×	×	×	×	×	×	×	×	×
Adverse events			×	×	×	×	×	×	×	×	×

*PRP* platelet-rich plasma,* PHN* postherpetic neuralgia,* NRS-11* Numeric Rating Scale (0–10) (measures pain intensity),* SF-12* 12-item Short-Form Health Survey (assesses quality of life),* PSQI* Pittsburgh Sleep Quality Index (evaluates sleep quality),* D* day,* W* week,* M* month

### Outcome Assessment

#### Baseline Demographic Characteristics

Baseline demographic characteristics of the patients will be collected through institutional electronic medical records or direct inquiry to compare with post-intervention outcomes.

#### Primary Outcome

Incidence rate of PHN: The incidence rate of PHN will be calculated based on the percentage of patients with an NRS score ≥ 4 at the follow-up visits scheduled at 3 months, 6 months, and 12 months.

#### Secondary Outcomes

1. Pain intensity: Assessed using the NRS-11, where scores range from 0 (no pain) to 10 (worst possible pain).
2. Pain characteristics: Including the presence of symptoms such as itching, paresthesia and hyperalgesia.
3. Skin lesions: Including the number of days from the initial onset of rash to healing, the largest skin lesion area.
4. Quality of life score: Assessed using the 12-item Short-Form Health Survey (SF-12) [29].
5. Sleep quality: Evaluated using the Pittsburgh Sleep Quality Index (PSQI) [30].
6. Analgesic consumption: Average weekly consumption and name of rescue analgesics for each patient.
7. Adverse events and safety assessment: Comprehensively record and analyze all treatment-related adverse events, including intra-treatment and post-treatment complications and adverse reactions.

### Statistical Analysis

All analyses will be conducted on an intention-to-treat (ITT) and per-protocol (PP) basis to ensure the robustness of the results.

#### Primary Outcome

A chi-square test will be used to compare the incidence of PHN between the PRP treatment group and the antiviral therapy group. Additionally, the relative risk (RR) and its 95% confidence interval will be calculated to evaluate the relative risk of PRP treatment compared to antiviral therapy.

#### Secondary Outcomes

For quantitative data (such as NRS-11, SF-12, and PSQI scores), the t-test or Mann–Whitney U test will be used to compare the differences between the two groups according to the data distribution characteristics. For categorical results (such as complication rate), chi-square test or Fisher's exact test will be used. All statistical analyses will use two-sided tests, with the significance level set to 0.05. For continuous repeated measurement data, such as quality of life scores at different time points after treatment, repeated measures ANOVA or mixed models will be used to analyze data for time effects and between-group effects.

#### Sample Size Calculation

Based on the reported prevalence, the incidence of PHN after antiviral therapy with famciclovir after VZV relapse in patients > 50 years of age is approximately 34.8%[31]. We predict a similar incidence rate in the control group. For the PRP group, our clinical experience suggests an estimated incidence of 10%. To achieve a 90% confidence level and a 5% significance level, we used PASS V.15 software (NCSS, Kaysville, UT, USA) to estimate that a minimum of 58 patients per group is required. Considering a potential 10% dropout rate, we plan to recruit 64 patients per group, totaling 128 patients.

#### Handling of Outliers and Missing Data

To address missing data, multiple imputation will be utilized. Outliers will be analyzed based on their origin and nature to determine whether correction, removal, or the application of data transformation methods is necessary to mitigate their impact.

#### Data Collection and Management

*Study conduct and data collection*: This study will be conducted by specially trained research assistants responsible for collecting data from patients who meet the inclusion criteria. We will employ an Electronic Data Capture (EDC) system to collect baseline data prior to treatment, including demographic characteristics, NRS scores, SF-12, and PSQI, among others. To ensure data accuracy and confidentiality, all data will be initially recorded on paper Case Report Forms (CRFs), then verified and entered into the EDC by data management personnel.

*DSMC review:* The Data Safety Monitoring Committee (DSMC) will conduct regular reviews of the data to ensure the safe progression of the study and promptly identify any potential safety issues.

*Data access and protection:* All researchers accessing the data are required to sign a confidentiality agreement and undergo training on data protection regulations.

*Adverse events (AEs):* AEs will be meticulously documented in the CRFs and assessed by researchers for causality with the treatment. The nature, timing, duration, severity, and outcome of AEs will be recorded and evaluated by physicians. All AEs will be promptly addressed and reported to the study monitor and Institutional Review Board (IRB), especially those that are serious or life-threatening. The aim is to address any issues promptly, including halting the study if necessary.

## Discussion

This study is a randomized, single-center, open-label, endpoint-blinded investigation aimed at evaluating the therapeutic effects of ultrasound-guided paravertebral PRP injection for alleviating acute ZAP and preventing PHN. Both ZAP and PHN significantly impair patients' sleep quality and emotional well-being [32]. Early therapeutic intervention is pivotal in reducing the incidence of PHN. However, even with the administration of tricyclic antidepressants, selective serotonin reuptake inhibitors, gabapentin, or pregabalin, a subset of patients may still necessitate additional treatment modalities to manage their symptoms effectively [33].

This study introduces a novel therapeutic strategy for ZAP: ultrasound-guided paravertebral injections of PRP. The concentrated release of growth factors from platelets within PRP facilitates tissue repair and regeneration. Consequently, PRP has found extensive applications across various medical fields, including sports medicine, orthopedics, neurology, endocrinology, and dermatology [34–37]. Utilizing autologous biological materials, PRP reduces the risk of systemic side effects caused by medications, which is particularly significant for patients reliant on long-term pain management drugs. We anticipate that the biological properties of PRP may similarly apply to neural tissue, thereby offering therapeutic benefits for ZAP and preventing the occurrence of PHN. From a clinical perspective, if PRP treatment proves to be effective, it could offer a new therapeutic option for patients with PHN who respond poorly to conventional therapies or cannot tolerate their side effects. Moreover, the successful implementation of PRP treatment may prompt medical professionals to re-evaluate existing treatment algorithms and incorporate it into standard treatment options, potentially improving overall patient quality of life and alleviating the psychological burden of chronic pain.

## Strengths and Limitations

Ultrasound-guided paravertebral PRP injections provide a minimally invasive approach for managing ZAP. By activating the body's natural healing, PRP may serve as an effective alternative to traditional antiviral and pain treatments, with fewer systemic side effects. This study could introduce a new option for alleviating ZAP and preventing PHN, enhancing patient quality of life and reducing reliance on medications.

However, certain limitations must be considered. Firstly, the quality of PRP preparation may vary due to technical aspects and operator proficiency, potentially affecting the consistency of therapeutic outcomes. Secondly, despite a sample size calculation ensuring adequate statistical power, the small sample size and geographical limitations of the study sites may restrict the generalizability of the results.

## Conclusions

This study protocol is designed to assess the efficacy and safety of ultrasound-guided PRP therapy in managing ZAP. Should the research hypothesis be validated, PRP could emerge as one of the viable methods for providing pain relief and enhancing the quality of life for patients suffering from ZAP.

## Ethics and Dissemination

This study adheres to the 2013 SPIRIT statement and strictly follows the Declaration of Helsinki. It has received approval from the ethics review committee of the participating hospital (Approval Number: 2024-KY(0504)). All participants are required to sign an informed consent form before enrollment, which details the purpose of the study, procedures, potential risks and benefits, as well as privacy protection measures. This clinical trial has been registered on the Chinese clinical trial registry platform (ChiCTR), https://www.chictr.org.cn/index.html (Registration Number: ChiCTR2400087248, Date of Registration: 2024–07-23). The results of the study will be disseminated through various channels, including peer-reviewed scientific journals and industry conferences. Research data will be made available to the scientific community, in accordance with data sharing policies and ensuring participant privacy, to support future scientific research.

### Trial Status

This plan has been approved by the Second Affiliated Hospital of Guangxi Medical University. We plan to begin trial recruitment on August 1, 2024. We estimate that the study will need to last 3 years to reach the expected target of 128 participants. Therefore, enrollment in the PROCESS trial is expected to be completed by August 31, 2027.
